# Functional parathyroid cyst in a patient with systemic lupus erythematosus: a case report

**DOI:** 10.1530/EDM-14-0100

**Published:** 2015-02-01

**Authors:** Jingjing Jiang, Mei Zhang, Ronghua He, Meiping Shen, Wei Liu

**Affiliations:** Department of Endocrinology, The First Affiliated Hospital of Nanjing Medical University, 300 Guangzhou Road, Nanjing, 210029, People's Republic of China; 1 Department of Endocrinology and Metabolism, Zhongshan Hospital, Fudan University, Shanghai, 200032, People's Republic of China; 2 Department of General Surgery, The First Affiliated Hospital of Nanjing Medical University, Nanjing, 210029, People's Republic of China; 3 Department of Nuclear Medicine, The First Affiliated Hospital of Nanjing Medical University, Nanjing, 210029, People's Republic of China

## Abstract

Functional parathyroid cysts are a rare cause of primary hyperparathyroidism and are often mistaken for thyroid cysts. Systemic lupus erythematosus (SLE) is also a very rare cause of hypercalcemia. We report the case of a 62-year-old woman, who was diagnosed with SLE 30 years ago, presenting with clinical and biochemical features of primary hyperparathyroidism. Laboratory investigation revealed increased serum calcium and parathyroid hormone (PTH) levels; neck ultrasonography (USG) revealed 40×34×26 mm cystic mass in the left lobe of thyroid gland. PTH level in the cysts was >2500 pg/ml, determined by USG-guided fine-needle aspiration (FNA). In this case, no evidence for potential pathogenic association between parathyroid cyst and SLE was uncovered. However, the recognition of this association is very important because the therapeutical strategy is completely different. Operative management is usually straightforward and alleviates symptoms and any biochemical abnormalities caused by the cyst.

**Learning points:**

- Functional parathyroid cysts are the rare cause of primary hyperparathyroidism and are often mistaken for thyroid cysts.
- SLE is also a very rare cause of hypercalcemia.
- Ultrasound-guided FNA of cystic fluid with assay for PTH level is an accurate method of differentiating parathyroid cyst from thyroid cyst.
- Appropriate management of functional parathyroid cysts is surgical excision.

## Background

Hypercalcemia is a disorder that most commonly results from malignancy or primary hyperparathyroidism. Functional parathyroid cysts are a rare cause of primary hyperparathyroidism (1) and are often mistaken for thyroid cysts (2). Parathyroid cysts have been clinically divided as either functional or nonfunctional (3) (4) (5), depending on their association with hyperparathyroidism.

Systemic lupus erythematosus (SLE) is a multi-system disorder with variable presentation. SLE is also a very rare cause of hypercalcemia. Up to now, about 300 cases of parathyroid cysts and ten cases of SLE patients presenting with hypercalcemia had been reported respectively in the literature. We reported a case of functional parathyroid cyst in a patient with previously diagnosed SLE.

## Case presentation

A 62-year-old woman was admitted to our hospital with a palpable mass in her left anterior neck, which was accidentally discovered 1 month ago. She had a 30-year history of SLE and was irregularly treated by prednisolone and *Tripterygium wilfordii*. She had a 10-year history of hypertension, which was well controlled by nitrendipine. Splenectomy was performed 10 years ago because of hypersplenism. On physical examination, a 4×3 cm mass was palpable in the left anterior neck, elastic and smooth was identified. She had erythema on her face and both hands. Velcro rales were heard at both lower lobes of the lungs. There was slight edema on both legs.

## Investigation

Initial laboratory tests disclosed the following values: serum calcium 3.55 mmol/l (normal, 2.15–2.65), phosphorous 1.04 mmol/l (normal, 0.86–1.86). Hemoglobin was 9.9 g/dl (normal, 11.0–16.0) while leukocytes and platelets were normal. 24-h urine calcium was 14.9 mmol (normal, 2.5–7.5). PTH (1–84) was 576 pg/ml (normal, 10–69), and osteocalcin 63.8 μg/l (normal, 11–43). Thyroid-stimulating hormone was 1.9 mIU/l (normal, 0.3–4.2), free thyroxine was 13.6 pmol/l (normal, 12.0–22), free triiodothyronine was 3.7 pmol/l (normal, 3.1–6.8). Calcitonin was 46.5 pg/ml (normal, 0–100) as well as tumor markers (AFP, CEA, CA50, CA19-9, CA72-4, CY21-1, NSE) were all normal. Antinuclear antibody level was 358 U/ml (normal, 0–40) and anti-dsDNA level was 39 U/ml (normal, 0–100). Anti-Sm antibody was also positive. C3 was 0.39 g/l (normal, 0.88–2.01) and C4 0.05 g/l (normal, 0.16–0.47). Erythrocyte sedimentation rate (ESR) was normal and rheumatoid factor (RF) was negative. Hyaluronic acid was 360 ng/ml (normal, 2–110), type IV collagen 154.2 ng/ml (normal, 19.8–112.8), and type III precollagen 213.6 ng/ml (normal, 0–120), suggesting hepatic fibrosis.

**Figure 1:**
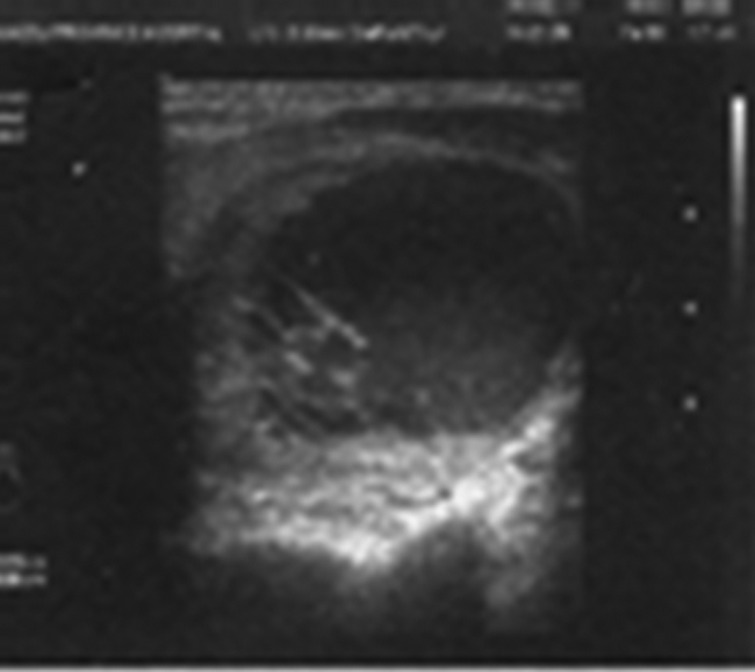
A sagittal ultrasound view demonstrated a 4.03 cm predominant cyst behind the left lobe of the thyroid gland.

**Figure 2:**
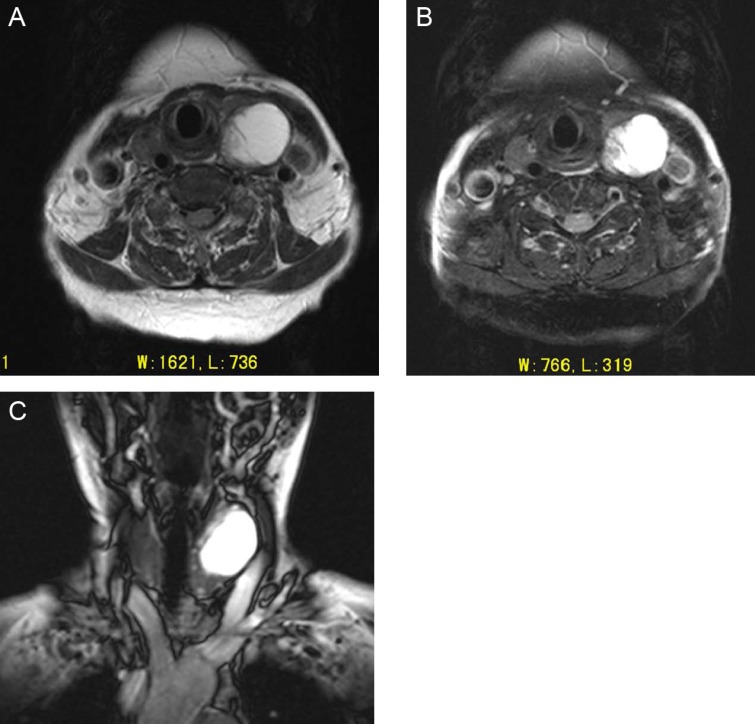
The cyst is located at the lower part of anterior neck behind the left lobe of the thyroid gland. High-intensity imaging of the content on both T1- (A) and T2- (B and C) weighted MRI suggested that the cyst area was filled with fluid.

**Figure 3:**
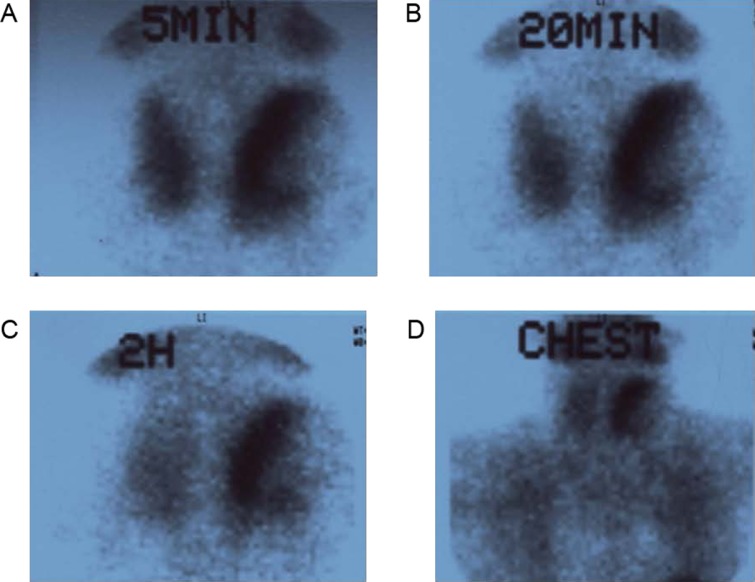
^99m^Tc-MIBI scintigraphy demonstrated focal accumulation and persistent uptake of radiotracers at the wall of the cystic mass. (A and B) Early images of 99mTc-MI; Panels C and D: delayed images of 99mTc-MIBI.

X-ray showed subperiosteal bone resorption in the middle and distal phalanges of the fingers. But no sign of osteoporosis was observed in skull, neck of femur, or lumbar spine. Spot-like shadows were seen at both lower lobes of the lungs on X-ray. Ultrasonography (USG) scan revealed a 40×34×26 mm cystic mass in the left lobe of thyroid gland (Fig. 1). High-intensity images on both T1 and T2-weighted magnetic resonance imaging (MRI) suggested that the cystic area was composed of liquid (Fig. 2). And USG scan of kidney and urinary tract revealed nephrolithiasis in the left kidney. Parathyroid scintigraphy using ^99m^Tc-MIBI showed intense accumulation and persistent uptake of radioactivities by the wall of the cyst (Fig. 3). Fine-needle aspiration (FNA) of the cyst was performed under ultrasound guidance, and 20 ml of bloody fluid was removed. The aspirate's PTH level was >2500 pg/ml. The mass shrinked immediately after aspiration, but reemerged overnight. Cytological analysis revealed a few macrophages and benign-appearing, follicular cells.

## Treatment

**Figure 4:**
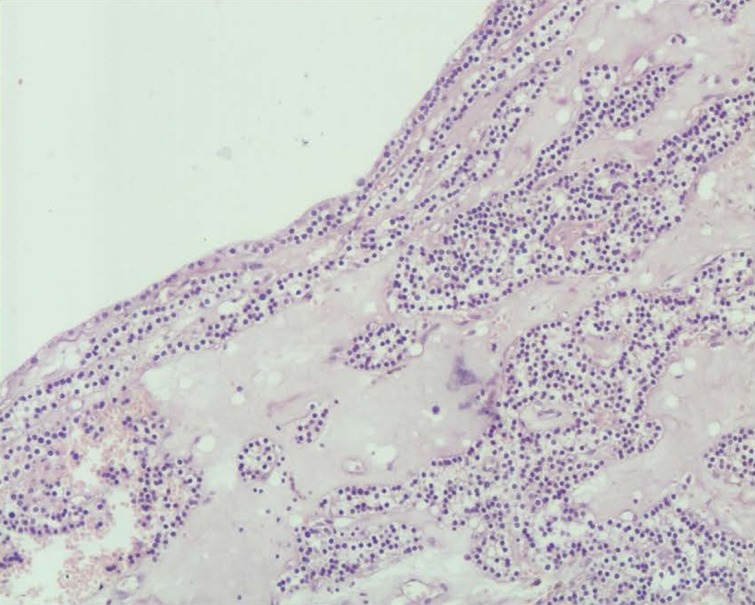
Parenchymal cells were arranged in trabecular pattern. The tissue contained abundant degenerative collagen fibers and blood vessels. There was no cellular atypia or capsular invasion (H&E, original magnification ×100).

Serum calcium persisted around 3 mmol/l despite treatment with oral alendronate (70 mg, qw) and calcitonin shot (200 IU, qd). Neck exploration was subsequently performed and 40×30 mm parathyroid cyst was surgically removed. Neither vascular invasion nor metastasis to local lymphnodes was observed. On the histology, parenchymal cells were arranged in trabecular pattern without cellular atypia or capsular invasion. The tissue contained abundant degenerative collagen fibers and blood vessels (Fig. 4). Histological examination verified a cystic adenoma of parathyroid gland.

After surgery, the patient complained of numbness of hands, which was relieved after taking calcium gluconate tablets (0.5 g, tid). Three days after surgery the patient left hospital, with serum PTH level being 19.9 pg/ml and serum calcium level being 2.29 mmol/l under oral substitution of calcium. SLE was treated with prednisolone. Thalidomide (50 mg, qn) was added to treat erythema, which subsided 1 month later. The patient is currently in good condition without any medication, but still suffered from SLE.

## Discussion

Hypercalcemia is a common electrolyte abnormality. More than 90% of cases of hypercalcemia are due to primary hyperparathyroidism or malignancy.

Macroscopic parathyroid cysts are rare clinical lesions, with <300 cases reported in the literature. These cysts are usually classified as either functional or nonfunctional based on serum PTH and calcium levels. Approximately one-third of parathyroid cysts are functional (3) (4) (5) and account for 1% of all cases of hyperparathyroidism (5). These cysts are more common in females than males (2.5:1) and usually located in an inferior gland with a left-sided predominance as in this case.

Parathyroid cysts account for <1% of neck masses and often present as a solitary nodule in the neck, posing a diagnostic challenge. USG, MRI, as well as scintigraphy, can provide helpful but limited information. Preoperative cyst fluid analysis is of great value for differential diagnosis (6) (7). The fluid from a parathyroid cyst is usually clear and colorless, containing elevated PTH level, though bloody fluid has also been reported (5) (8). Ultrasound-guided FNA of cystic fluid with assay for PTH level is an accurate method of differentiating parathyroid cyst from thyroid cyst. In the present case, the content was bloody with extremely high levels of PTH, confirming the diagnosis of a functional parathyroid cyst. High level of tumor antigen-like CA19-9 in cyst content has been previously reported (8). However, analysis of the cyst fluid revealed no elevation of either CA19-9 or other tumor markers in this case.

SLE is a very rare cause of hypercalcemia, with only ten cases reported (9) (10). The pathogenesis involves the presence of stimulatory anti-PTH receptor autoantibodies or excessive endogenous production of PTHrP primarily due to lymphadenopathy (10) (11). Interestingly, SLE was also found to coexist with hypoparathyroidism, with four cases reported in literature (12). The underlying pathophysiology was supposed to be either a common genetic predisposition or the extension of the autoimmune process to the parathyroid glands, which is still under debate (12).

In this presented case, SLE was stable and could be safely ruled out as a major cause of hypercalcemia because PTH level was very high and serum calcium;eve; normalized soon after surgery. Two cases have been previously reported of primary hyperparathyroidism and SLE in a single patient (13) (14). Together with this case, all three patients were women aged over 40 years with previously diagnosed SLE. They were all treated with corticosteroids and the conditions of SLE were relatively stable with anti-dsDNA normal in two patients. Postoperatively, all patients were relieved of hypercalcemia.

The pathogenesis of parathyroid cysts varies from degeneration of a parathyroid adenoma (infarction within the adenoma), coalescence of microcyst with hypersecretion within the capsule of the gland, or persistent embryological remnants of branchial cleft cysts (15). No involvement of autoimmunity in hyperparathyroidism has been suggested in literature. Considering the relative frequency of these two diseases in a general population, especially in middle-aged women, this coexistence cannot be ruled out as a coincidence.

In this case, no evidence for potential pathogenic association between parathyroid cyst and SLE was uncovered. However, the recognition of this association is still important because the therapeutic strategy is completely different.

## Patient consent

Written informed consent was obtained from the patient for publication of this case report.

## Author contribution statement

All co-authors listed contributed substantially to the preparation of this manuscript.
